# An Insight into Emerging Begomoviruses and their Satellite Complex causing Papaya Leaf Curl Disease

**DOI:** 10.2174/1389202924666230207111530

**Published:** 2023-06-23

**Authors:** Aarshi Srivastava, Vineeta Pandey, Abdullah. M. Al-Sadi, Muhammad S. Shahid, R.K. Gaur

**Affiliations:** 1 Department of Biotechnology, D.D.U. Gorakhpur University, Gorakhpur, India;; 2 Department of Plant Sciences, College of Agricultural and Marine Sciences, Sultan Qaboos University, Al-Khod, Oman

**Keywords:** Papaya leaf curl disease, begomovirus, evolution, diversity, management, *Bemisia tabaci*

## Abstract

Papaya leaf curl disease (PaLCD) was primarily detected in India and causes major economic damage to agriculture crops grown globally, seriously threatening food security. Begomoviruses are communicated by the vector *Bemisia tabaci*, and their transmission efficiency and persistence in the vector are the highest, exhibiting the widest host range due to adaptation and evolution. Symptoms induced during PaLCD include leaf curl, leaf yellowing, interveinal chlorosis, and reduced fruit quality and yield. Consequently, plants have evolved several multi-layered defense mechanisms to resist Begomovirus infection and distribution. Subsequently, Begomovirus genomes organise circular ssDNA of size ~2.5–2.7 kb of overlapping viral transcripts and carry six–seven ORFs encoding multifunctional proteins, which are precisely evolved by the viruses to maintain the genome-constraint and develop complex but integrated interactions with a variety of host components to expand and facilitate successful infection cycles, *i.e*., suppression of host defense strategies. Geographical distribution is continuing to increase due to the advent and evolution of new Begomoviruses, and sweep to new regions is a future scenario. This review summarizes the current information on the biological functions of papaya-infecting Begomoviruses and their encoded proteins in transmission through vectors and modulating host-mediated responses, which may improve our understanding of how to challenge these significant plant viruses by revealing new information on the development of antiviral approaches against Begomoviruses associated with PaLCD.

## 1 INTRODUCTION

### 1.1 Global Epidemics, and Geographical Distribution of Begomovirus Infection in Papaya

The papaya plant (*Carica papaya* L.), a member of the Caricaceae family, is an economically valuable crop with heavily nutritional and therapeutic benefits [1]. It is consumed fresh in salads or with curries. They are commercially valued in the juice processing, cosmetic, leather, pharmaceutical, and food industries [2]. Papaya leaves have traditionally been used to treat thrombocytopenia associated with dengue fever [3]. For hundreds of years, people have consumed papaya leaves for a variety of therapeutic purposes. The demand for papaya has steadily expanded in the pharmaceutical and healthcare industries in recent years as researchers have discovered its various therapeutic potentials.

The papaya plant originally developed in Southern Mexico and Central America. India and many other countries, such as South Africa, Sri Lanka, *etc.*, are currently cultivating it. Andhra Pradesh is the leading papaya-growing state, followed by Maharashtra, Madhya Pradesh, Gujarat, and Karnataka. The papaya crop has been subjected to various biotic and abiotic challenges throughout the years, the most prominent of which is PaLCD, which is primarily liable for the massive loss in papaya production. Thomas and Krishnaswamy initially described PaLCD in 1939, and the causal pathogen was later identified [4]. However, several virus diseases significantly threaten crop production, with Begomoviruses significantly limiting Papaya cultivation and production worldwide [5]. Meanwhile, several Begomovirus species have been spreading around the world, massively increasing their host range. Since then, the number of Begomoviruses affecting papaya plants has risen steadily. The association of Begomovirus diversity with PaLCD has been described in India, causing serious production losses [6]. The rapid adaptation behaviour of Begomoviruses, causing evolutionary peril to papaya production.

**Fig. (1):**
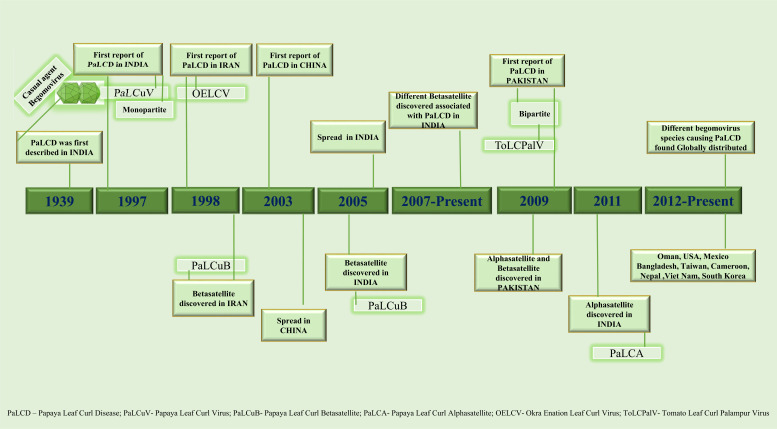
Origin and history of Papaya Leaf Curl Disease (PaLCD) (Begomoviruses and associated sub-viral satellites).

**Fig. (2):**
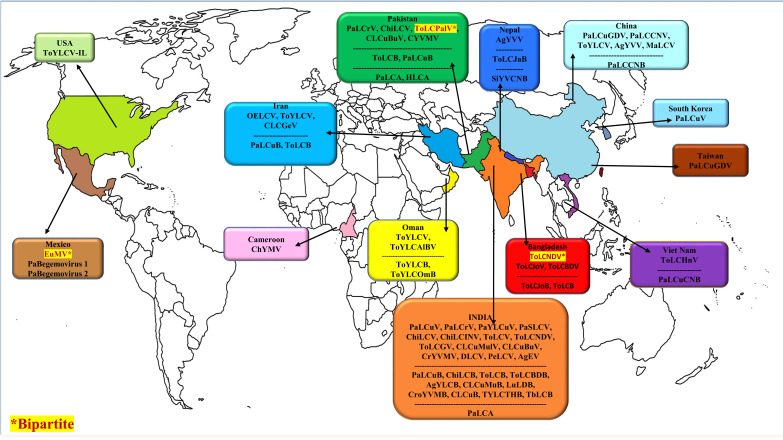
Worldwide distribution of Begomovirus species associated with Papaya Leaf Curl Disease.

PaLCD is triggered by a group of monopartite Begomoviruses (family Geminiviridae) whose species are communicated in nature by the vector *B. tabaci* (order Hemiptera, family Aleyrodidae) in a persistent, quasi, and circulative means [7, 8]. This pest has a global distribution and is a major concern for many agricultural crops. Throughout its lifespan, it resides underneath the host plants, feeds on the leaves, and harms them both directly and indirectly by piercing them and sucking out the sap, as well as by changing the host plant's development, photosynthesis, and phytochemical processes [9]. It is a complex of various genetically distinct species, the majority of which frequently develop over a large number of generations and may quickly assimilate pesticide resistance, consequently making their control difficult for many economically valuable crops [10, 11]. Papaya leaf curl virus (PaLCuV) is an economically significant Begomovirus that has been documented to cause devastating damage to papaya plants, and it is more prevalent in northeast India [6]. Among viral diseases, PaLCD triggered by a complex of Begomovirus species is a chief barrier to papaya production, influencing overall papaya production in the Indian subcontinent [6, 12]. Epidemics of Begomoviruses with associated betasatellites and alphasatellite that cause PaLCD were not limited to Asian countries; they were also identified in American and African countries. It has been found that several Begomoviruses infect papaya plants globally; their origin and association with sub-viral satellites are depicted in Fig. (**1**) (Data from NCBI). However, Begomoviruses associated with PaLCD have been rapidly expanding their host range and dispersal to different geographical regions in recent years. The considerable diversity of Begomovirus distressing papaya plants in India suggests homological diversification, where intra- and inter-species relations were observed forming different major and minor linages with their closely related species. Similar cases were observed for the betasatellite population associated with PaLCD [6]. However, to better understand the prevalence of Begomoviruses infecting Papaya plants, a map (Fig. **2**) is presented, depicting the evolution of Begomovirus species over time (Fig. **1**) related to leaf curl disease of *C. papaya*.

## 2 VIRUS–VECTOR INTERACTIONS

Begomoviruses are communicated by the vector *B. tabaci* under natural conditions. The whitefly is profoundly polyphagous vector, and because it is globally spread and difficult to sustain, it is categorized as a supervector [13]. Whitefly is a multi-biotype that includes biotypes Q, B, and A, which are presently classed as Mediterranean, Middle East Asia Minor 1, and New World species, respectively. Kollenberg *et al*. 2014, [14] found that transmission efficiencies vary greatly between these biotypes and even within intra-species populations. The prevalence of Begomovirus in both cultivated and wild plants over the last 20 years is thought to be related to the worldwide dispersal of the former two, *i.e*., biotypes Q and B, of *B. tabaci* species. For millions of years, it appears that Begomoviruses and their vectors have coevolved [15]. This allows viral diversity to spread to new hosts and the emergence of new viruses [16], making these insects an ideal platform for disseminating viral communities in plants across a region [17]. Whitefly may imitate and endure throughout the year in wild plants in tropical and subtropical locations, resulting in rapid proliferation and dominance in agro-ecosystems, which can lead to significant epidemics of Begomovirus diseases in overlaying crops [18].

Begomovirus infection was shown to alter the vector feeding behaviour and promote plant fitness and attraction, thus ascertaining an indirect mutualistic relationship between virus and vector, thereby boosting vector performance, and increasing pathogen distribution in the host. The virus can be conveyed by a single whitefly, and females have a higher transmission efficiency than males. The finding shows that Begomovirus genes and proteins interact closely with those of the *B. tabaci*, and that essential protein receptors are present in whitefly's gut that allow viral entry into the insect haemolymph *via* endocytosis, allowing virus discharge into the host plant *via* salivary secretion [19]. Even though the prevalence of biotypes Q and B is mostly found in association with Begomovirus disease, the expansion and emergence were found to be significantly mediated by the B biotype vector because of its greater survival and fecundity [20]. Thus, due to direct vector manipulation by the virus, altered feeding behaviour, and indirect vector affinity for Begomovirus-infected plants, this mutualistic interaction favours the co-evolution of both the vector and the virus. There is no such report that solely explains the role of a specific whitefly biotype in relation to PaLCD. Moreover, recent research reveals that now the scenario is more sophisticated, with biotypes varying in transmission efficiency, competing, and replacing one another, and engaging mutually with Begomoviruses. It will be of interest to determine the factors in Begomovirus-infected papaya plants that are detrimental to one biotype but favourable to another biotype and to identify whether mutualism prevails in other *B. tabaci*–Begomovirus associations or not. The impact of such mutualism would help investigate Begomovirus prevalence and diversity [13].

The virus–vector relationship was studied, and several genes were found that revealed a persistent circulative transmission mode of Begomovirus in *B. tabaci*. Most of them, when downregulated, reduce the movement and transmission of Begomovirus, while others, upon upregulation, facilitate virus translocation in the digestive tract and ease transmission [21] (Table **S1**). Genes operating the quantity of Begomovirus in whiteflies and the efficiency of virus dissemination were studied in a few species of Begomovirus, such as Tomato leaf curl virus (ToLCV), Tomato yellow leaf curl virus (TYLCV), Tomato yellow leaf curl China virus (ToYLCNV), and Cotton leaf curl virus (CLCuV) [22]. However, despite these recent developments, the proteins influencing the virus movement within the whitefly body and along the direction followed by virions are still not known in the case of PaLCuV. Furthermore, vector adaptation to advanced farming methods and climatic change are critical factors in the genesis of new plant virus diseases. Global warming will affect the epidemiology of Begomovirus-related plant diseases by modifying the circulation of vectors and their host range [23]. As a result of mixed infections, novel combinations of geminiviruses and satellites may emerge faster, potentially expanding the host range and circulation of geminiviruses. For the reasons stated above, as well as continued vector movement among both crop and wild plants and repetitive mixed virus infections, it is highly probable that we will see further outbreaks in the years ahead. As a result, continuous monitoring of vectors and the geminiviruses they carry appears to be necessary for long-term virus management.

## 3 GENOME STRUCTURE AND PROTEIN FUNCTIONS

### 3.1 Viral Genes and Proteins

Begomovirus associated with PaLCD are primarily monopartite Begomovirus with a closed circular ssDNA encapsidated in a quasi-isometric non-enveloped twinned particle of ~ 2.8 kb size comprising a virus genome. The nature of the genome can either be monopartite (single component DNA-A) between 2.5-3.1 kb or bipartite (two similar-sized components DNA-A & DNA-B), each between 2.6 and 2.8 kb [24]. Even though in bipartite Begomovirus, DNA-A can duplicate autonomously, nuclear localization requires DNA-B components. Monopartite Begomovirus and DNA-A of bipartite Begomovirus are homologs and encode for six proteins; two proteins, such as coat protein (AV1/ CP) and pre-coat protein (AV2/ pre-CP) are encoded on the virion sense strand, while four proteins, such as transcription activator protein (AC2/TrAP), replication enhancer protein (AC3/REn) replication-associated protein (AC1/Rep), C4(AC4) protein on complementary sense strand [25]. Begomovirus DNA-B encodes for Movement protein (MP) and nuclear shuttle protein (NSP) for local (nucleus to cytoplasm) and systemic (cell to cell) movement, respectively [26]. Thus, mutually they trigger systemic infection and develop characteristic symptoms [27]. An intergenic region (IR) separates the reading frames of both the DNA-A and DNA-B components of a bipartite Begomovirus, which preserves the integrity of the bipartite genome and allocates a common region (CR) within the intergenic region that contains a conserved stem-loop structure with a nonanucleotide (TAATATT/AC) sequence and replication origin (ori), which are clearly recognized by the Rep protein of viruses. Although there is uncertain knowledge that clearly supports the means through which multipartite and segmented genomes have arisen, some suggestions imply that they may have developed by the disintegration of the genome of their non-segmented ancestors, with imperfect segments becoming contagious by complementation [28]. In context, Sivalingam *et al*. 2012 [29], suggested that the DNA-B of bipartite could have developed from a satellite molecule caught by the monopartite progenitor of all Begomoviruses. Possibly, this pattern offered superior flexibility to the monopartite ancestors, and consequently, it was continued over the Begomovirus evolutionary process. Moreover, the exceptionality of monopartite Begomovirus is the existence of small ssDNA satellite molecules that significantly boost the virus virulence, thus named as alphasatellite and betasatellite molecules of sizes ~1.4 kb and ~1.3 kb, respectively, and in a few cases, a newly reported deltasatellite molecules are found consociated with Begomovirus [30, 31].

### 3.2 Plant Defense Strategies

Plants have developed a surveillance system that prevents infections from surviving due to frequent exposure to a wide spectrum of pathogens. To cope with virus attacks, antimicrobial peptides (AMPs) and pathogenesis-related (PR) proteins are stimulated in plants under abiotic and biotic stresses [32]. Pattern recognition receptors (PRRs) are special plant receptors that recognise pathogen-associated structure and protein (molecular patterns) and stimulate pathogen-associated molecular-PTI (pattern-triggered-immunity) in plants. Similarly, a different group of resistance (R) proteins stimulates ETI (effector-triggered immunity) in plants by identifying the pathogen’s secreted microbial proteins [32, 33]. The PAMP-PRRs complex, along with associated co-factors such as SERKs (Somatic Embryogenesis Receptor-Like Kinase), and SOBIR1 (Suppressor of BIR1-1), creates a cascade of intracellular incidents such as oxidative burst, ion influx, stimulation of MAPKs (Mitogen-Activated Protein Kinases), and increased defense hormone biosynthesis, which ultimately leads to resistance responses in plants [34, 35]. Simultaneously, ETI and PTI result in SAR (systemic acquired resistance), the development of resistance in healthy and distant tissues [36]. Moreover, RNA silencing, a defense mechanism based on a particular sequence that can target viral as well as cellular mRNA for degradation, is one of the evolutionary conserved surveillance systems in plants. The technique involves the methylation of the virus DNA, which prevents replication by preventing transcription. Another mechanism is the hypersensitive response defense system, which, when activated by infection, releases chemical substances that prevent infection by destroying infected cells [37]. The hormone SA (salicylic acid) has a crucial function, it inhibits NPR1-NPR3 (Non-Expressor of Pathogen Related Genes) and promotes interaction between NPR1andNPR4, which induces the expression of PR (Pathogen Related) genes, which is essential for the development of SAR [35, 36]. However, the host's most particular defensive mechanisms are triggered, conferring resistance during viral disease.

Resistance genes (R-genes) and RNA silencing-mediated defense mechanisms are two widely studied reactions in plants towards viral disease. These techniques are based on genetic engineering, which has permitted the introduction of specified genes that protect plants from abiotic and biotic stresses, as well as viral infection. The functional characterization of certain of the gene's loci reveals that they play a role in RNAi-mediated host defense [38]. Crop improvement includes gene transfer methods that use gene resistance loci for disease resistance, and many initiatives have been found for successful commercial cultivation of transgenic papaya that combat papaya ringspot virus [39], but such efficient management is not available for PaLCuV.

### 3.3 Suppression of Plant Defenses

**Fig. (3):**
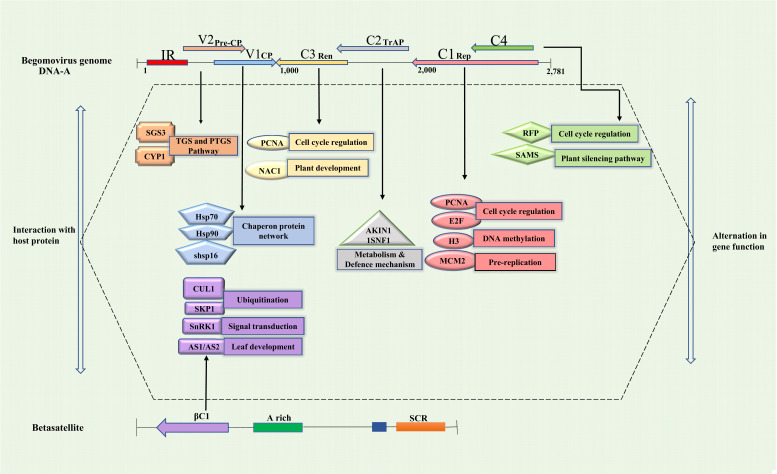
Crucial role of viral encoded proteins in pathogenicity during host-virus interaction (modulating RNAi, a host protective mechanism).

Suppression of plant defenses is crucial for the progress of virus infection, and respond to host defense, viruses are fortified with RNA silencing suppressor (RSS) activity that blocks the distribution of silencing signals, and encodes viral suppressors of gene silencing (VSRs) that regulate pathogenicity. The genomic organisation and operational features of the six viral proteins of Begomovirus associated with PaLCD indicate that most of the proteins are multifunctional. The expression and function of neighbouring proteins are closely regulated by overlapping and embedded ORFs in the genome, which weakens the host defense with the help of VSRs and establishes the efficient replication, packaging, and dissemination of viruses in the host. Viruses successfully cleave various classes of sRNAs, such as long dsRNA, ss-siRNA, and ds-siRNA, to establish disease in the host. Furthermore, VSRs also inactivate various other proteins involved in RNA silencing pathways and block the systemic spread and cell-to-cell movement of sRNA silencing [40]. Begomoviruses encode various VSRs, and their systematic activity in suppressing plant RNAi machinery is summarized in Table **S2**. Begomoviruses have emerged to develop multifunctional proteins that help them regulate their life cycle and inhibit host defense. Pre-coat, Coat protein, Rep, TrAP, REn, C4, and βC1 proteins are crucial viral proteins encoded by different ORFs that have a role in pathogenicity during host-virus interaction, most possibly by modulating RNAi, a host protective mechanism (Fig. **3**).

## 4 BIOLOGY AND INTERACTION OF SATELLITE MOLECULES

### 4.1 Disease Severity and Host Range

India, a prominent papaya-yielding country in the world, accounts for a serious infection impact of Begomovirus species, which hinders viable papaya production and, more importantly, could slow down the expansion of the pharmaceutical industry. As a result, Begomovirus infection associated with PaLCD causes significant damage to the papaya crop, which endured not only biochemical and anatomical losses, but also a significant loss in its pharmacological property [41]. Begomoviruses are distributed all throughout the world, but they are more prevalent in areas with warm-temperate climates and a wide diversity of crops. The vector whitefly is linked to the spreading of Begomovirus and has a vast host range, as it is a direct pest of many crops. Desolation of the affected plant has been attributed to a varied range of dicotyledonous plants in subtropical and tropical regions, particularly in the families Solanaceae (Chilli, Tomato), Malvaceae (Okra, Cotton), Leguminosae (Mungbean, Dolichos), Fabaceae (Soybean), Euphorbiaceae (Cassava, Jatropha), Cucurbitaceae (Pumpkin, Cucumber), Caricaceae (Papaya), and Brassicaceae (Radish) [42].

Papaya plants infected with Begomoviruses exhibit distinctive symptoms, including crinkling of leaves, downward or upward curling yellow mottling, vein clearing, leaf blistering, and puckering of leaves. Plants seem stunted with condensed internodes with reduced fruit setting in the later stages of infection, ultimately resulting in full crop loss [43]. In natural infections, satellite DNAs may coexist with Begomovirus infecting papaya plants and modulate its pathogenicity, including its host range. Betasatellites are typically associated with Begomoviruses that infect papaya plants [44]. The replication and distribution of these DNA satellites are reliant on the helper virus and are recognized to code for βC1 protein that has been related to disease, most likely due to its capacity to suppress plant defense systems interrelated to gene silencing [45]. Conversely, the mixed infection of several viral species, high genetic diversity, a high evolution rate, and the advent of new strains or species [6] may establish a big challenge in developing resistance to PaLCD. Due to the possible importance of betasatellites associated with monopartite Begomovirus during PaLCD, its diversity is also considered in this review (Fig. **2**).

### 4.2 Interaction of Satellite Molecules

In the field, monopartite “Old World” Begomovirus species are frequently found to be related to DNA satellite molecules such as betasatellites and alphasatellites [25]. Although the combination of an alphasatellite with PaLCD was rarely observed, betasatellite was found to be highly prevalent in India [6]. The biology and interactions of satellite molecules associated with PaLCD have been explored; however, rare evidence is found regarding the occurrence of these molecules in endemics. In subsequent years, various Begomovirus species were observed to infect papaya plants. Studies indicated the association of a few alphasatellites and various betasatellites in widely held interactions, suggesting the involvement of these molecules in increased disease severity and a wide host range. The correlation of Begomovirus with betasatellites assists in the virus's systemic transmission and increases virus accumulation in the host plant. This demonstrates that betasatellites acquired through mixed infection with multiple Begomoviruses in a tolerant host may benefit Begomovirus pathogenicity. Following that, a study discovered that Tomato leaf curl New Delhi virus (ToLCNDV) interacts with betasatellites such as isolates of croton yellow vein mosaic betasatellite and radish leaf curl betasatellite, resulting in radish and tomato leaf curl disease. Therefore, it indicates that betasatellites have a role in promoting the ToLCNDV host range. Furthermore, ToLCNDV association with other Begomoviruses DNA-B and betasatellite resulted in the loss of natural resistance in chilies [46].

A diverse study of PaLCD also involves Begomovirus-associated satellites, which highlights the emergence and evolution of Begomovirus. This includes papaya leaf curl betasatellite, croton yellow vein mosaic betasatellite, tomato leaf curl java betasatellite, and tomato leaf curl betasatellite from different countries [47]. However, infection with variable Begomoviruses on the same crop demonstrates the coexistence and wide host selection of several betasatellites. This is a mixed infection, which has made organizing management strategies difficult, as multiple Begomovirus infections are found with papaya plants, making them a reservoir and alternative host for many Begomoviruses. Phylogenetic analysis helps detect sequence diversity among the virus isolates. The immense range of dispersal assists in comprehending the viruses' origin, dispersal, evolution, and etiology of diseases, which would contribute to developing viral disease management strategies.

## 5 HOST ADAPTION AND EVOLUTION

Since the 1990s, humans adapting to modernisation have been found to influence aspects of the appearance and progression of many geminivirus diseases. Recombination and mutation, such as transversion substitution and non-synonymous mutation, have been found to be key players in evolutionary outcomes that differ according to the host plant, type of virus, inoculated plant age, and inoculum homogeneity. For recombination, the common region (CR) of DNA A acts as a hot spot point, which not only gets exchanged between member species but also with heterologous molecules, *i.e*., DNA-B component or betasatellites. In 2007, Idris [48] and colleagues developed a reassortment model by exchanging segments of DNA-A and DNA-B between different Begomoviruses, which concluded that test hosts are less virulent than corresponding wild-type viruses, indicating that adaptive modulation of virulence is strongly intertwined. Additionally, Nawaz-ul-Rehman in 2009 [49] described the adaptation behaviour of Begomovirus by showing recombination events between the common regions (CR) of DNA-A and DNA-B, which are consequently gaining more virulence. As a result, the reassortment combination thatoccurred within the Begomovirus genome significantly contributes to a diverse and novel host range. Synergistic interactions have been observed between an unrelated satellite and a specific virus, with the ability to alter and enhance the virus's host range and pathogenicity [50]. Evidence was reported from Brazil by Silva and associates, in 2014 [51], which showed the recombination between two genes, *i.e*., the rep gene and the CR region, causes Begomovirus adaptation. The method involves a biolistic inoculation of those genes from Tomato ringspot virus (ToRSV) and Tobacco mosaic virus (ToRMV), thus resulting in the emergence of ToRMV having pseudo-recombination and recombination, therefore highlighting the significance of recombination in adaptation and the emergence of Begomoviruses. Moreover, point mutations and betasatellite induced trans-replication might contribute as well.

Variable pathogenicity for Begomoviruses may be found depending on the host plant, with some causing obvious symptoms and accumulating to considerably high titres in specific hosts while infecting others only infrequently. According to previous research, the quantity of virus accumulation and degree of symptom expansion may be determined by the coordinated expression of host defense reactions in each plant species [52]. The ability of a virus to infect and cause symptoms in each host plant is determined by the expression of specific genes and the effective interactions between virus- encoded proteins and the host plant. The genomes of Begomoviruses may be implicated in host adaptation through coding and non-coding regions [53].

A vulnerable host delivers all the obligatory aspects for virus transcription, replication, and progress, while a quasi-host prevents the virus from multiplying and spreading due to incompatible interactions between viral factors and the host. Plants have transformed in response to pathogen attacks to activate a coordinated activity of multiple layers of defense. Begomoviruses are recognized to encode numerous viral suppressors of RNA silencing or viral determinants that permit the overpowering of plant resistance features to counteract plant defense [54]. The efficacy of infection and host adaptation capacity will be determined by the interplay between the host plant's defense response to disease and the Begomovirus' capacity to impede this defense response. Different responses to infection may develop in Begomovirus- infected host plants, such as susceptible and resistant cultivars of the same plant species [55]. In this regard, it is worth noting that virus resistance to plant defenses varies from one viral isolate to the next and even within isolates of the same strain [54]. Restricted genetic changes might be liable for Begomoviruses' significant variations in host adaptability. It should also be noted that the interaction of satellite DNAs with Begomoviruses, particularly betasatellites, may modify the virus's pathogenesis in a single host plant [56] or possibly contribute to generating systemic infections and expanding the virus's host range, as seen in other geminiviruses [29]. Perhaps it could be associated with the role of RNA silencing processes in plant defenses against Begomoviruses, as well as the betasatellites' powerful suppressor of gene silencing. As a result, the existence of betasatellites may have a role in Begomovirus host adaptability.

### 5.1 Infectious Plant Materials and Agricultural Trades

One of the utmost significant aspects leading to the global distribution of viruses/insect pests is the movement of agricultural products. Several geminiviruses have been found to reach new agroecological zones around the world as a result of human activity. For instance, Tomato yellow leaf curl Thailand virus has been traced to Myanmar, Thailand, and China [57]. Also, a bipartite Begomovirus watermelon chlorotic stunt virus (WmCSV): An Eastern Hemisphere Begomovirus (spread across Eastern Mediterranean countries) introduced into the Western Hemisphere [58]. Likewise, infected plants from Europe are responsible for the appearance of Tomato Yellow Leaf Curl China Virus (TYLCV) in France and Spain [59]. Similarly, the emergence of the TYLCV in America is traced to infected plants being transported from Israel [60].

### 5.2 Climate Condition

In recent decades, environmental change, which has been considerably facilitated by climate variations, has had a significant impact on the dispersion and distribution of viruses, thereby delivering appropriate circumstances for disease/vector proliferation. A variation in environmental conditions can expand insect vector populations drastically [57]. Conversely, little rainfall and temperatures between 25 and 33°C promote an increase in the whitefly population, whereas cold temperatures and excessive rainfall suppress the whitefly population. Dusty storms and heavy winds aid in the long-distance journey of whiteflies, accelerating the spread of viruses [61].

### 5.3 Reassortment (Pseudo-recombination)

During mixed infections, pseudo-recombination is common in bipartite Begomoviruses and involves the exchange of viral components, allowing them to increase their virulence and infect a greater number of hosts. This is mostly achieved with the same Begomovirus isolates rather than distinct Begomovirus species [37]. As a result, a report describes the reassortment of bipartite and monopartite Begomoviruses, which results in the acquisition of the DNA- B component and the transformation of monopartite Begomoviruses into bipartite Begomoviruses [62]. Further, Singh and co-workers in 2008 demonstrated that the reassortment between the components of ToLCNDV and ToLCGV caused more serious damage in tomatoes than their counterparts, and the same pseudo-recombination, when observed in Chili, results in virus resistance breakdown [46]. Thus, for a greater understanding of the evolution of plant viruses, we need a framework such as population genetic structure, phylogenetic relationships, adaptive evolution events, past demographic histories, and molecular datasets to monitor the changes in virus populations, which might help to design the appropriate control measures.

### 5.4 Recombination

Recombination explains the exchange and incorporation of fragments during replication from an RNA or DNA strand into a diverse individual strand. This replication process represents the normal evolutionary mechanism of viruses through recombination–driven processes rather than an exceptional one [63]. The process can be both intra and interspecific. This genetic recombination has been found to play a dynamic role in the emergence of the most devastating phytopathogens Geminiviruses, specifically the Begomoviruses [64]. The functional necessity for interaction between Begomovirus and its satellite can be quite informal. Therefore, the combination of new Begomovirus- satellites with components from variable geographic regions is quite feasible, which may overwhelm plant defense machinery and enhance the host range of Begomoviruses. The evolutionary dynamics and host-virus interaction show that the DNA-A and DNA-B components respond differently to evolutionary events, with DNA-B being more tolerant to recombination and variation than DNA-A. In field work, isolates of multiple bipartite Begomoviruses, such as Chili Leaf Curl Virus (ChiLCV), Bhendi Yellow Vein Mosaic Virus (BhYVM), CLCuV, and several Tomato Leaf Curl-Begomoviruses, and betasatellites, have frequently been found in mixed infections [46, 65]. Mixed infections undoubtedly promote the emergence of novel recombinants with the ability to expand their host range [66]. Molecular variation is thought to occur among Begomoviruses as a result of recombination between different DNA-A components, resulting in virulence for recombination [49]. Genetic recombination is a critical mechanism in the emergence of various virus families, and it has been significantly documented for associates of the Geminiviridae family [67]. It allows parental viruses in mixed infections to exchange genetic information and extrapolates their progeny through parasexual reproduction. Recombination exchanges occur frequently in Begomoviruses at both the intraspecies and interspecies levels. There are many cases of interspecific recombination reported, which greatly improved the understanding of predicting recombination events with RDP software [68]. Nine methods are prescribed in the RDP software used for analysing datasets. These are PhylPro, Maxchi, LARD, GENECONV, RDP, Bootscan, SiScan, Chimera, and 3Seq [69]. To determine the positive result of recombination events, seven out of nine methods or five out of seven methods, or three out of five methods are used. Begomoviruses and their satellite molecules actively participate in recombination, hence acting as a major driving force in the emergence of severe disease complexes caused by Begomoviruses [70]. However, a study on recombination analysis associated with PaLCD displayed the role of variable major and minor parents in causing an uneven distribution of recombination breakpoints, thus indicating their importance to genetic diversity. The Rep gene region of the Begomovirus population reflects the highest number of recombination break points within nucleotide positions 1,527–2,612, followed by the CP gene, and the least number of recombination break points were observed in the C4 genome region. The distribution of events was maximally detected among intra-species isolates, and the least was found among inter-species isolates. Similar recombination events were discovered to be primarily distributed in the βC1 genome region of betasatellites causing PaLCD, supporting the prevalence of recombination that is involved in virus movement by suppressing host antiviral silencing genes [6]. It is worth noting that adaptive recombination in geminiviruses may be exceedingly efficient. Recombinant Begomoviruses show considerable changes in host adaptation, even causing catastrophic epidemics, implying that recombination is a mechanism for evolution and adaptation to changing environmental conditions [71].

### 5.5 Mutation

Rapid evolution can be spotted in viruses in real time due to the result of changes in genetic information, which they use to interact within and with the environment. Thus, genetic information can be a driving force in understanding and investigating the changes in genotypic information causing evolution [72]. Environmental factors such as chemical substances or the involvement of UV light in base modification, as well as the incorporation of DNA or RNA Polymerase into non-complementary nucleotides, escape the cellular repair mechanism, altering genotype, resulting in a point mutation. However, the question of what caused the emergence of more than 400 Begomoviruses, despite having the similar genomic organization to old world and new world Begomoviruses apart from AV2 (pre-CP) ORF in the new world Begomoviruses representing the same origin regardless of distribution, arises. Moreover, most of the transition mutation types are commonly observed as substitution mutations rather than insertions or deletions, which depend on inoculum homogeneity, inoculated plant age, virus type, and host plant. However, most recent reports indicate that some (+) ssDNA and ssRNA viruses do not use the usual host mechanism for mismatch repair, resulting in substitution rates that act as a driving force accelerating the emergence, evolution, and maintenance of novel Begomovirus species [73]. As a result, the rates of transitional, transversional DNA substitution mutation for the Begomoviruses and associated betasatellites causing PaLCD were studied, and it was discovered that the CP and Rep genes of genomic DNA-A is more prone to transitional and transversional substitution mutations, respectively, and accept more frequent amino acid changes than other genomic regions. Therefore, the study suggests that the role of base substitutions causes transition mutation at a higher rate in the CP region and transversion mutation at a higher rate in the Rep region of the Begomovirus population associated with PaLCD. Additionally, the mean substitution rate for the Indian Begomovirus and betasatellite populations explained the high mean substitution rate working at wobble codon position C3 of genomic regions, leading to codon degeneracy and genetic variation within and among populations [6].

## 6 STRATEGIES FOR CONTROLLING PaLCD DISEASE

The PaLCD is a terror to all papaya plant-producing countries where whitefly is predominant as a major or minor pest. Several management strategies were devised to control Begomovirus-associated diseases, such as performing short-term management practices and developing long-term resistant varieties either through non-conventional or conventional means.

### 6.1 Short Term Strategies

After the spread of the Begomovirus-associated disease at an extensive scale, it is preferable to confine the size of the whitefly population. A few immediate measures were established to control the Begomovirus-associated disease, including dropping the vector whitefly population in the field, expending pesticides on different crops, eliminating weeds, and adopting healthy plant cultivation practices through the delivery of stable doses of biological agents, fertilizers, *etc*. [74].

The PaLCD is not seed-borne. It endures on alternate hosts such as soybean, tomato, chilli, okra, hibiscus, ageratum, and tobacco, *etc*., showing leaf curl-like symptoms. Weeds may aid in preserving the pools of inoculants for viruses [15]. In general, the elimination of weeds from the crop field cuts the possibility of accessibility by another host, thus reducing the dormant initiator of inoculum. Pure cultivation is imperative for regulating the whitefly population and infection frequency, either through the application of weedicides or by cultural practices. Cultural practices involve the intercropping of various aromatic plants such as coriander and basil, whose secondary metabolites act as impervious, which have been stated to deliver an assured degree of defense against whitefly infestation in tomato plants [75]. In India, farmers normally grow vegetables, oil seed crops, fruits, and fodder, which act as alternative hosts for the virus and provide an amiable environment for plant viruses to make genetic recombination with added viruses and evolve as novel strains of the virus. Thus, forbidding the cultivation of alternate hosts in the close area of any other crop and evading the farming of other crops in the off-season would help in monitoring the vector population by interrupting the lifecycle of the whitefly [76].

In Pakistan, the application of chemicals is based on a certain threshold level, which recommends 4-5 whiteflies per leaf for achieving the density and size of the whitefly population, although a single virulent whitefly can transmit virus from one plant to another. The application of selective insecticides was shown to a certain degree to control the whitefly population, which not only preserved the population of predators but also delayed the development of chemical resistance in the whitefly population [77]. In this regard, cultivators constantly treat seeds with insecticides followed by spraying chemicals that specify protection from whitefly infestation up to 45 DAS or, in some articles, up to 75 DAS. The effectiveness of this method is being studied on cotton plants, which allows the cotton plant to evade the disease, thus reducing damage. The treatment of biopesticides is an additional control approach to managing the whitefly population [78]. Nonetheless, its impact on papaya plants is yet to be comprehended. The impairment of PaLCD can also be diminished by the application of a suitable quantity of fertilizers. The use of excessive nitrogenous fertilizers reduces disease resistance; on the other hand, the use of potassium can increase disease resistance, most likely due to its role in maintaining an energy gradient, osmoregulation, and molecular compound synthesis. It also impacts the metabolic function of the host by working on the compatibility relationship between the parasite and host [79]. As a result, it is suggested that a stabilized proportion of potassium and nitrogen fertilizer can help reduce disease austerity. It is resolved that by regulating the whitefly populations by biological, cultural, and chemical means, only partial eradication of the disease is probable since a small whitefly population may result in disease spread and prevalence.

### 6.2 Long-term Strategies

#### 6.2.1 Host Plant Genetic Resistance

When infection occurs frequently and repeatedly throughout the season, the only effective method is to breed counteracting crop varieties. For designing breeding strategies, the genetic basis of resistance and its inheritance are crucial constituents for developing virus-resistant crop varieties and have been recommended as a long-lasting approach to managing the disease and its causal agents effectively [80]. The variation in incidence and severity of the disease rests on the whitefly population, virus titer, and host genetic makeup. In a period of 350 million years, various plant species have established defense mechanisms by synthesising secondary metabolites and toxic compounds to counter vectors, viruses, and associated diseases. Earlier, it was assumed that resistance towards causal agents of disease is because of multiple environmental factors, including host plant, plant age, light, relative humidity, temperature, *etc*., that may influence the disease’s occurrence and severity [80].

Li *et al*., 2016 [81] reported virus-induced gene silencing (VIGS) in *G. hirsutum.* However, silencing of Mitogen-activated protein kinase (GhMPK3) resulted in suppression of the ethylene (ET), WRKY-JA (jasmonic acid), and MPK pathways, which improved whitefly sensitivity. Thus, these genes can be used to increase host plant resistance [81]. Under natural or controlled conditions, multiple efforts are required to recognize resistant sources for PaLCD by inspecting the material, either through grafting of diseased buds or by exposing the papaya plants to virulent whiteflies. However, in the case of cotton plants [82], most resistant genotypes are photoperiod susceptible, which can be widely used in refining the cotton varieties and germplasm for the CLCuD. Moreover, cytological reports of some resistant plants revealed successful introgression of resistant genes [83]. A few virus-infection-resistant crop varieties were established using recombination breeding methods, and this case study is still needed for PaLCD.

#### 6.2.2 Genetic Approaches for Improving Resistance

Genetic tools are crucial in establishing gene function, expression, and their regulation, tagging genes of interest, developing genetic linkage maps, and creating transgenic plants. In linkage mapping, the interspace between a DNA marker and a trait is estimated [84]. The use of DNA markers in rising resistant cultivars is very important because the recognized DNA markers can be used in introducing marker-assisted selection (MAS). Many researcher’s studies showed how to map crop’s associated diseases and their causal agents using resistance QTLs between inter- and intra-specific crosses. In a study to detect DNA markers associated with genes conferring resistance to the disease, a random amplified polymorphic DNA (RAPD) assay and RFLP markers were employed [82, 85].

#### 6.2.3 Use of Transgenic Approaches

**Fig. (4):**
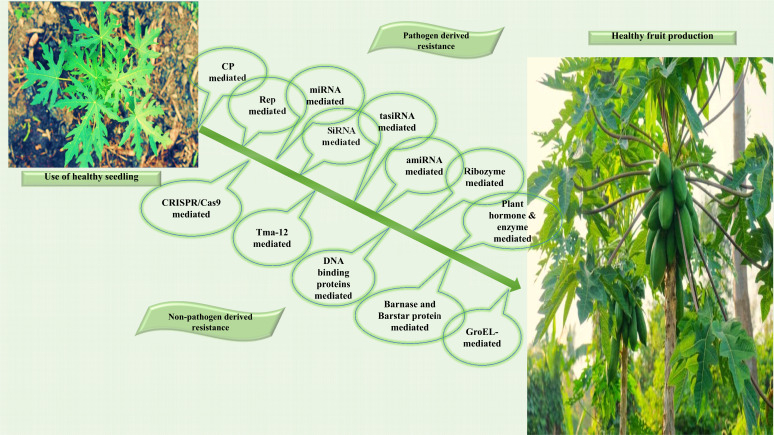
Biotechnological approaches towards management of Papaya leaf curl disease.

Traditional methods have most of the limitations for managing diseases caused by Begomovirus, but the use of genetically modified resistant variants has been found to be the most sustainable and efficient for controlling virus infections. Several genetic engineering strategies have been used to improve crop varieties by conferring resistance to virus- associated diseases. This development advances biotechnology and bio-agriculture, which enables the use of *Agrobacterium tumefaciens* for developing transgenic plants and agrichemicals, the production of hybrid seed, modern plant breeding, and farm mechanisation to provide viral control and nutrient management. These strategies are basically based on using genes from other, indirectly related genetic sources (non-pathogen derived), and full-length genes or diverse small, conserved portions of the virus (pathogen-derived resistance) [85] (Fig. **4**).

Consequently, pathogen-derived resistance is achieved in varietyof plants, providing resistance against Begomoviruses. Employing genomic knowledge of the viruses, the following strategies have been used to develop resistance [86]. This includes Coat Protein (CP)-Mediated Resistance, which has been shown to be effective against a variety of DNA and RNA viruses. This method advances our understanding of the task of capsid protein in virus encapsulation and cell-to-cell movement in infected plants, paving the way for the generation of transgenic plants [28]. A protein-derived resistance provides resistance to Begomoviruses by using a AC1-expressed gene known as Rep gene. Moreover, to control infection caused by Begomoviruses, virologists employed the microRNAs (miRNAs) mediated resistance mechanism, which is present in plants as small noncoding RNA molecules and regulates the expression of genes. Further, small RNA (siRNA) mediated resistance provides a natural immunity against multiple Begomoviruses by regulating the expression of various genes in all plants [87]. This method uses siRNA-based genetic engineering (SRGE) technology, which has been found to be an effective tool for crop protection. Another defense mechanism used by plants against Begomovirus infections is known as artificial trans-acting siRNA, which uses the phenomenon of post-transcriptional gene silencing to repress the viral gene. This tasiRNA has been found to provide resistance against AV2, AC2, and AC4 proteins of geminiviruses. Singh and co-workers, in 2015 [88], used the mechanism of tasiRNA for the first time to provide virus resistance. Additionally, Antisense RNA ribosomes mediated resistance strategy against virus infection, which uses a mechanism of intrinsic endonucleolytic activity that cleaves the target RNA and controls antisense dominance. Antisense

RNA technology uses the antisense RNA molecule and functions by suppressing the targeted complementary mRNA and by inhibiting its expression. This study was conducted in transgenic cotton for Begomoviruses in order to direct the virus's Rep and CP genes, which suppressed viral replication, movement, and encapsidation [89]. RNAi is primarily based on transcriptional gene silencing (TGS) and post-transcriptional gene silencing (PTGS) and is used to examine the function of genes for providing resistance to viral diseases in a variety of crop species [90], including those found effective against Mung Bean Yellow Mosaic Virus (MYMV), African Cassava Mosaic Virus (ACMV), and CLCuV [91]. Another method used to provide resistance against Begomovirus disease is the artificial-miRNAs (amiRNA) mediated resistance strategy, which alters the target specificity and has important impacts on biotic and abiotic stress, signal transduction, and plant development. However, when the V2 gene of CLCuBuV was used as an amiRNA construct and tested for its reaction in a model species, *Nicotiana benthamiana*, the results were asymptomatic transgenic plants [92]. Regarding the above transgenics tool, Plant hormones and enzymes also play a crucial role in developing pathogen-derived resistance. In plants, the main defense hormone is jasmonic acid, which is effective in controlling necrotrophic pathogens and herbivorous insects. In a recent study, it was demonstrated that jasmonic acid has a critical task in assembling resistance to insects in new plants of Arabidopsis, which is modulated by miR156-targeted-SPL9 (negatively correlated with JA expression) [93]. Subsequently, a well-established phenomenon explains the interaction of host proteins with viruses, which leads to overpowering the host protein gene. Further investigation into this interaction has revealed that the CLCuMuV-associated pathogenicity determinant βC1 protein gene of betasatellites interacts with the host's Ubiquitin conjugating (E2) enzyme S1UBC3, resulting in βC1 overexpression, which overpowers the cumulation level of polyubiquitinated proteins in transgenic plants [94].

The other liberal approach to providing significant resistance against viruses uses a plant gene-based, non-pathogen-derived resistance. Because of the increased rate of plant virus infection, many conventional and non-conventional strategies for controlling rapidly emerging and evolving plant viruses have been developed. Towards this scenario, the most systematic, reproducible, and simple technology used for the Genome Editing Approach is clustered frequently interspaced palindrome repeats-associated 9 (CRISPR/Cas9) systems which have developed many resistance varieties against Begomoviruses [95]. Apart from transcription activator-like effector nucleases and zinc finger (ZFN), the more precise CRISPR/Cas9 system has piqued the interest of scientists from all major fields of science, particularly plant biologists, as a promising genome editing tool. Furthermore, because of its robustness, adaptability, and ease of engineering, it has the potential to control Begomoviruses [96]. In bacteria, the CRISPR/Cas9 system confers immunity to intruding nucleic acids. The CRISPR spacers significantly cut the invading DNA molecules. The resulting molecules (20 nt long) are analogous to those produced by RNAi. These are found in ~40% of sequenced bacterial genomes and 90% of sequenced archaeal genomes [97]. The CRISPR/Cas9 system has been used to edit genomes in a variety of complex organisms by providing Cas9 protein and guiding RNAs into cells. Numerous loci can also be addressed utilising various sgRNAs technologies [98]. CRISPR has been used to build resistance to geminiviruses. For instance, the genome of the Bean yellow dwarf virus (BeYDV) was modified in beans using the CRISPR–Cass system, resulting in reduced virus multiplication in the host and lowered disease symptoms [99]. CRISPR/Cas9 technologies have also been demonstrated to reduce TYLCV disease symptoms [100]. A sgRNA–Cas9 construct was also used to produce resistance to the beet severe curly top virus in *Nicotiana benthamiana* [101]. This approach has been considered one of the approaches for controlling geminiviruses [102]. The genome engineering strategy has recently emerged as a promising tool for developing desirable traits in plants. Rather than the causative agent, genes from the host or non-host plant are employed to engineer disease resistance. Plants have been genetically modified to produce coat-binding proteins, DNA-binding proteins, antiviral antibodies, and other genes that give resistance to viruses and their pathogenicity [103]. Recently, in fern, a protein called Tma12 has been discovered that imparts whitefly resistance. Cotton Coker 312 was transformed with the gene encoding this protein. One transgenic cotton strain has shown improved resistance to whiteflies (>99%), and in rats, this protein was found to be non-toxic. As a result, this gene could be employed to control whitefly populations in papaya plant and in added crop species in the future [104]. To the best of our knowledge, such programs and further experiments are needed to be exploited to combat the vector whitefly population and viruses associated with PaLCD.

For limiting the proliferation of geminivirus in transgenic plants, cell death induction has been used. It was attained by the mutual activity of Barstar and Barnase proteins from *Bacillus amyloliquefaciens*. Barnase is a ribonuclease (RNase), and barstar prevents it from working. If geminivirus infection is not present, the two transgenes should express at the same levels to prevent RNase synthesis. This method was used to suppress the ToLCNDV, and the virus's sweep to other tissues was restricted [105]. Moreover, whitefly population suppression in transgenic tobacco plants encoding insecticidal genes under the phloem promoter has recently been described [106]. However, control of Begomoviruses in papaya plants is yet to be realized.

Another method uses DNA-binding proteins, *i.e*., artificial zinc finger (AZP), to develop virus resistance by only binding to viral DNA sequences and not host DNA. This protein targets the Rep-specific direct repeats of the v-ori of invasive geminiviruses and inhibits viral replication. This method has been effectively shown in wheat, rice, sunflower, and other plants that have numerous ZF domains in their resistant genes [107]. However, the effectiveness of this method in suppressing geminiviruses in papaya plants has yet to be demonstrated. Similarly, an alternative genome editing tool that could be used is TALEN. It is made up of a non-specific FokI nuclease domain united to a programmable DNA binding domain, as well as a DNA-binding domain that contains conserved repeats obtained from transcription activator-like effector proteins (TALEs). TALEs could modify the transcription of genes in the host cell [108]. This incident can also be exploited to control PaLCD.

GroEL-mediated protection was also studied toward effective resistance to numerous virus species. The GroEL protein, which is produced by a bacterium in the whitefly's gut, binds to the CP of Begomoviruses, causing the viruses to be destroyed in the vector’s haemolymph [109]. This tool can be used to develop resistance to a variety of viruses from various taxonomic genera. For example, the GroEL gene from *B. tabaci* was transferred into a transgenic tomato and protected it from TYLCV [110]. The GroEL protein has been linked to the conduction of TYLCV and potato leaf roll virus (PLRV) by *B. tabaci* and aphids, respectively [111]. The efficacy of these proteins to inhibit PaLCD and its causal agents must be investigated.

## CONCLUSION

Reduced agricultural yields of commercially important crops all over the world are a result of geminivirus-infested plants that are spread by insects. Begomovirus, the most common genus, is responsible for 50 to 90 percent of crop losses and is on the rise globally. A finite number of viruses (genomes) are accumulated by the vector *B. tabaci* as a result of the plant’s constant feeding on Begomovirus-infected plants, typically within 12 to 48 hours [112]. This process also increases the vectors’ survival, high transmission rates, and wide range of host-virus interactions, causing the disease to persistently infect new and fresh crops every day. Begomoviruses are unable to encode enough proteins since their tiny genome prevents them from completing their infectious cycle. Moreover, most of these versatile viral proteins could effectively control various cellular machinery and serve as factors that determine pathogenicity. Plants deploy a variety of defense mechanisms to prevent geminiviruses from spreading by preventing favourable conditions for virus growth and limiting the spread of the virus. As a result, geminiviruses activate host genes, interact with plant proteins, and/or modify the cell cycle in order to interfere with host growth and hormonal processes and decrease host-mediated defense responses. This permits the harmonious co-evolution of viruses and plants. Additionally, as the characteristics of a pathosystem are critical for defining plant infection by certain viruses, it is crucial to examine the elements that influence virus infections between resistant and susceptible cultivars. In order to regulate geminivirus infections, it is necessary to integrate all indirect and direct plant-virus interactions. Begomovirus disease management has advanced significantly as a result of intensive research into the biology and epidemiology of Begomovirus diseases. As agricultural technology advances, more and more molecular methods are needed to prevent geminivirus infection and crop loss. It may be possible to use molecular methods like RNA interference (RNAi), genome editing approaches, and other pathogen-derived and non-pathogen-derived resistance approaches, which were reviewed, to minimize the prevalence of several Begomovirus diseases. These methods are effectively used for managing diseases, enhancing crops, and creating extensive quarantine plans.

## LIST OF ABBREVIATIONS

ACMV: African Cassava Mosaic Virus
AMPs: Antimicrobial Peptides
AZP: Artificial Zinc Finger
BeYDV: Bean Yellow Dwarf Virus
BhYVM: Bhendi Yellow Vein Mosaic Virus
ChiLCV: Chili Leaf Curl Virus
CLCuD: Cotton Leaf Curl Disease
CLCuMuV: Cotton Leaf Curl Multan Virus
CLCuV: Cotton Leaf Curl Virus
CP: Coat Protein
CR: Common Region
CRISPR/Cas9: Clustered Frequently Interspaced Palindrome Repeats-Associated 9
DNA: Deoxyribonucleic Acid
ET: Ethylene
ETI: Effector-Triggered Immunity
GhMPK: Mitogen Activated Protein Kinase
IR: Intergenic Region
MAPKs: Mitogen-Activated Protein Kinases
MAS: Marker-Assisted Selection
MP: Movement protein
MPK: Mitogen-Activated Protein Kinase
MYMV: Mung Bean Yellow Mosaic Virus
NCBI: National Center for Biotechnology Information
NPR: Non-Expressor of Pathogen
NSP: Nuclear shuttle protein
ORFs: Open Reading Frames
PaLCD: Papaya Leaf Curl Disease
PaLCuV: Papaya Leaf Curl Virus
PAMP: Pathogen-Associate Molecular Patterns
PLRV: Potato Leaf Roll Virus
PR: Pathogen Related
PR: Pathogenesis-Related
pre-CP: Pre-Coat Protein
PRRs: Pattern Recognition Receptors
PTGS: Post-Transcriptional Gene Silencing
PTI: Pattern-Triggered Immunity
QTLs: Quantitative Trait Locus
R: Resistance
RAPD: Random Amplified Polymorphic DNA
RDP: Recombination Detection Program
REn: Replication Enhancer Protein
Rep: Replication-associated Protein
RFLP: Restriction Fragment Length Polymorphism
RNA: Ribonucleic Acid
RNAi: RNA Interference
RSS: RNA Silencing Suppressor
SA: Salicylic Acid
SAR: Systemic Acquired Resistance
SERKs: Somatic Embryogenesis Receptor-Like Kinase
SOBIR1: Suppressor of BIR1-1
SRGE: siRNA-Based Genetic Engineering
TALEN: Transcription Activator-Like Effector Nucleases
TALEs: Transcription Activator-Like Effector Proteins
TGS: Transcriptional Gene Silencing
ToLCGV: Tomato Leaf Curl Gujarat Virus
ToLCNDV: Tomato Leaf Curl New Delhi Virus
ToLCV: Tomato Leaf Curl Virus
ToRMV: Tobacco Mosaic Virus
ToRSV: Tomato Ringspot Virus
ToYLCNV: Tomato Yellow Leaf Curl China Virus
TrAP: Transcription activator protein
TYLCV: Tomato Yellow Leaf Curl China Virus
TYLCV: Tomato Yellow Leaf Curl Virus
UV: Ultraviolet
VSRs: Viral Suppressors of Gene Silencing
WmCSV: Watermelon Chlorotic Stunt Virus
WRKY-JA: Jasmonic Acid
ZFN: Zinc Finger
