# Supplementary material for: An Insight into Emerging Begomoviruses and their Satellite Complex causing Papaya Leaf Curl Disease
Source: Curr Genomics. 2023 Jun 23;24(1):2–17. doi: 10.2174/1389202924666230207111530 (PMC10334704; doi:10.2174/1389202924666230207111530)
Supplement: Supplementary file 1 [file CG-24-2_SD1.pdf]

Supplementary Material

An Insight into Emerging Begomoviruses and Their Satellite Complex causing Papaya Leaf Curl Disease

Aarshi Srivastava[1,$], Vineeta Pandey[1,$], Abdullah. M. Al-Sadi[2], Muhammad S. Shahid[2,*] and R.K. Gaur[1,*]

[1]*Department of Biotechnology, D.D.U. Gorakhpur University, Gorakhpur, India;* [2]*Department of Plant Sciences, College of Agricultural and Marine Sciences, Sultan Qaboos University, Al-Khod, Oman*

Table S1. Known genes regulating the amount and efficacy of Begomovirus transmission in *B. tabaci*.

**Gene**	***Acronym***	**Action site**	**Consequence**	**Reference**
**Defensin**	*BtDef*	Expressed in<br>➢ Salivary gland<br>➢ Midgut<br>➢ Fat bodies<br>➢ Ovaries	➢ DsRna-silencing lowers the large quantity expression of begomovirus in midgut.	[1]
**Cyclophilin B**	*CypB*	Expressed in<br>➢ Salivary gland<br>➢ Midgut	➢ Oral feeding with CypB antibodies reduces the begomovirus transmission	[2]
**Knottin-1**	*Knot-1*	Expressed in<br>➢ Abdomen	➢ Controls the amount of virus.<br>➢ DsRna-silencing accelerates the ingested quantity of virus and transmitted to host(plant) by various order of magnitude.	[3]
**Peptidoglycan recognition protein**	*BtPGRP*	Co-localized with begomovirus in midgut	➢ Up-regulated upon ingestion of begomovirus.	[4, 5]
**HSP70**	*HSP70*	Expressed in<br>➢ Midgut epithelial cells<br>➢ Co-localized with begomovirus in midgut	➢ Controls or inhibit virus transmission.<br>➢ Oral feeding with *HSP70* antibodies enhances the begomovirus transmission	[6]
**Midgut protein**	*MGP*	Expressed in<br>➢ Midgut	➢ Interacts with Coat protein of virus to ease transmission.	[7]
**Components of clathrin-mediated endocytosis**	*CME*	Expressed in<br>➢ Midgut barrier	➢ Transport of viruses transversely the gut barrier into the haemolymph	[8]

Table S2. Mechanistic action of various Begomoviruses-encoded RNA silencing suppressor protein (VSRs) to counter RNAi-mediated host defense.

Begomovirus(es)	VSRs	Regulate Host Biology	Reference
African casava mosaic virus	AC2	Interacts with transcriptional gene silencing	[9]
Tomato golden mosaic virus	AC2	Prevents transcriptional gene silencing and inactivates adenosine kinase	[10, 11]
Tomato yellow leaf curl virus	AC2	Interacts with transcriptional gene silencing	[9]
	V2	Unites with Suppressor of gene silencing3(SGS3) and inhibits its secondary siRNA production	[12]
	C4	Inhibits intercellular circulation of siRNA	[13]
Tomato yellow leaf curl China virus	βC1	Supresses S-adenosyl-homocysteine-hydrolase and transcriptional gene silencing;<br>Degrades SGS3 and stimulates Rgs-CaM (regulator of gene silencing calmodulin-like protein), thus inhibit secondary siRNA production	[14, 15]
Tomato leaf curl New Delhi virus	AC4	Cleaves Argonautes4 and affects transcriptional gene silencing	[16]
Mungbean yellow mosaic virus	AC4	Sequesters and supresses systemic movement of siRNA	[17]
Mungbean yellow mosaic India virus	AC5	Represses the expression of CHH cytosine transferase, thus obstruct RNA-directed DNA methylation	[18]
	AC2	Interferes with RNA dependent RNA polymerase6 and Argonautes1	[19]
Cotton leaf curl Multan virus	C4	Interferes with S-adenosyl methionine synthetase and inhibits its transcriptional gene silencing	[20]

References

[1] Wang, Z.Z.; Bing, X.L.; Liu, S.S.; Chen, X.X. RNA interference of an antimicrobial peptide, Btdef, reduces tomato yellow leaf curl China virus accumulation in the whitefly Bemisia tabaci. Pest Manag. Sci, **2017**, 73, 1421–1427. doi: 10.1002/ps.4472.

[2] Kanakala, S.; Ghanim, M. Implication of the whitefly Bemisia tabaci Cyclophilin B protein in the transmission of tomato yellow leaf curl virus. Front. Plant Sci, **2016**, 7, 1702. doi: 10.3389/fpls.2016.01702.

[3] Hariton-Shalev, A.; Sobol, I.; Ghanim, M.; Liu, S.S.; Czosnek, H. The Whitefly Bemisia tabaci knottin-1 gene is implicated in regulating the quantity of tomato yellow leaf curl virus ingested and transmitted by the insect. Viruses, **2016**, 8, 205. doi: 10.3390/v8070205.

[4] Wang, Z.Z.; Shi, M.; Huang, Y.C.; Wang, X.W.; Stanley, D.; Chen, X.X. A peptidoglycan recognition protein acts in whitefly (Bemisia tabaci) immunity and involves in Begomovirus acquisition. Sci. Rep, **2016a**, 6, 37806. doi: 10.1038/srep37806.

[5] Wang, L.L.; Wang, X.R.; Wei, X.M.; Huang, H.; Wu, J.X.; Chen, X.X.; Liu, S.S.; Wang, X.W. The autophagy pathway participates in resistance to tomato yellow leaf curl virus infection in whiteflies. Autophagy, **2016b**, 12, 1–15 doi: 10.1080/15548627.2016.1192749.

[6] G€otz, M.; Popovski, S.; Kollenberg, M.; Gorovits, R.; Brown, J.K.; Cicero, J.; Czosnek, H.; Winter, S.; Ghanim, M. Implication of Bemisia tabaci heat shock protein in begomovirus whitefly interactions. J. Virol. **2012**, 84, 13241–13252 doi: 10.1128/JVI.00880-12.

[7] Rana, V.P.; Popli, S.; Saurav, G.K.; Raina, H.S.; Chaubey, R.; Ramamurthy, V.V.; Rajagopal, R. A Bemisia tabaci midgut protein interacts with begomoviruses and plays a role in virus transmission. Cell. Microbiol, **2016**, 18, 663–678. doi: 10.1111/cmi.12538.

[8] Pan, L.L.; Chen, Q.F.; Zhao, J.J.; Guo, T.; Wang, X.; Hariton-Shalev, A.; et al. Clathrin-mediated endocytosis is involved in tomato yellow leaf curl virus transport across the midgut barrier of its whitefly vector. Virolology, **2017**, 502, 152–159. doi: 10.1016/j.virol.2016.12.029.

[9] Trinks, D.; Rajeswaran, R.; Shivaprasad, P.V.; Akbergenov, R.; Oakeley, E.J.; Veluthambi, K.; et al. Suppression of RNA silencing by a geminivirus nuclear protein, AC2, correlates with transactivation of host genes. J Virol, **2005**, 79(4), 2517-27. doi: 10.1128/JVI.79.4.2517-2527.2005.

[10] Wang, H.; Buckley, K.J.; Yang. X.; Buchmann, R.C.; Bisaro, D.M. Adenosine kinase inhibition and suppression of RNA silencing by geminivirus AL2 and L2 proteins. J Virol, **2005**, 79(12), 7410-8. doi: 10.1128/JVI.79.12.7410-7418.2005.

[11] Buchmann, R.C.; Asad, S.; Wolf, J.N.; Mohannath, G.; Bisaro, D.M. Geminivirus AL2 and L2 proteins suppress transcriptional gene silencing and cause genome-wide reductions in cytosine methylation. J Virol **2009**, 83(10), 5005-13. doi: 10.1128/JVI.01771-08.

[12] Glick, E.; Zrachya, A.; Levy, Y.; Mett, A.; Gidoni, D.; Belausov, E.; Citovsky, V.; Gafni, Y. Interaction with host SGS3 is required for suppression of RNA silencing by tomato yellow leaf curl virus V2 protein. Proc Natl Acad Sci U S A, **2008**, 105(1), 157-61. doi: 10.1073/pnas.0709036105.

[13] Rosas-Diaz, T.; Zhang, D.; Fan, P.; Wang, L.; Ding, X.; Jiang, Y.; et al. A virus-targeted plant receptor-like kinase promotes cell-to-cell spread of RNAi. Proc Natl Acad Sci U S A, **2018**, 115(6), 1388-1393. doi: 10.1073/pnas.1715556115.

[14] Yang, X.; Xie, Y.; Raja, P.; Li, S.; Wolf, JN.; Shen, Q.; et al. Suppression of Methylation-Mediated Transcriptional Gene Silencing by βC1-SAHH Protein Interaction during Geminivirus-Betasatellite Infection. PLoS Pathog, **2011**, 7(10), e1002329. doi: 10.1371/journal.ppat.1002329.

[15] Li, F.; Zhao, N.; Li, Z.; Xu, X.; Wang, Y.; Yang, X.; et al. A calmodulin-like protein suppresses RNA silencing and promotes geminivirus infection by degrading SGS3 via the autophagy pathway in *Nicotiana benthamiana*. PLoS Pathog, **2017**, 13(2), e1006213. doi: 10.1371/journal.ppat.1006213.

[16] Vinutha, T.; Gaurav, K.; Varsha. G.; Tomas, C.; Peter. P,; Ramesh, S.V.; Shelly, P. Tomato geminivirus encoded RNAi suppressor protein, AC4 interacts with host AGO4 and precludes viral DNA methylation. Gene, **2018**, 678, 184-195. doi: 10.1016/j.gene.2018.08.009.

[17] Carluccio AV, Prigigallo MI, Rosas-Diaz T, Lozano-Duran R, Stavolone L. S□acylation mediates Mungbean yellow mosaic virus AC4 localization to the plasma membrane and in turns gene silencing suppression. PLoS Pathog, **2018**, 14(8), e1007207. doi:10.1371/jo urnal.ppat.1007207.

[18] Li, F.; Xu, X.; Huang, C.; Gu, Z.; Cao, L.; Hu, T.; Ding, M.; Li, Z.; Zhou, X. The AC5 protein encoded by Mungbean yellow mosaic India virus is a pathogenicity determinant that suppresses RNA silencing-based antiviral defenses. New Phytol, **2015**, 208(2), 555-69. doi:10.1111/nph.13473.

[19] Kumar, R.V.; Singh, A.K., Singh, A.K.; Yadav, T.; Basu, S.; Kushwaha, N.; Chattopadhyay, B.; Chakraborty, S. Complexity of begomovirus and betasatellite populations associated with chilli leaf curl disease in India. J. Gen. Virol, **2015**, 96, 3157–3172. doi:10.1099/jgv.0.000254.

[20] Ismayil, A.; Haxim, Y.; Wang, Y.; Li, H.; Qian, L.; Han, T.; et al. *Cotton Leaf Curl Multan virus* C4 protein suppresses both transcriptional and post-transcriptional gene silencing by interacting with SAM synthetase. PLoS Pathog, **2018**, 14(8), e1007282. doi: 10.1371/journal.ppat.1007282.
